# Cardioprotective Effects of HuoxueAnshen Recipe against Myocardial Injuries Induced by Sleep Deprivation in Rats

**DOI:** 10.1155/2017/7359760

**Published:** 2017-04-05

**Authors:** Rong Yuan, Li-li Guo, Heng-wen Chen, Jun-ping Li, Zhong Chen, Ben-jun Wei, Jie Wang

**Affiliations:** ^1^Department of Cardiology, Guang'anmen Hospital, China Academy of Chinese Medical Sciences, Beixiange 5, Xicheng District, Beijing 100053, China; ^2^Clinical Medical College, Beijing University of Chinese Medicine, North 3rd Ring East Road No. 11, Chaoyang District, Beijing 100029, China; ^3^Laboratory of Cardiovascular Disease, Xiyuan Hospital, China Academy of Chinese Medical Sciences, Haidian District, Beijing 100091, China

## Abstract

*Background*. Traditional Chinese Medicine is extensively used in China and HuoxueAnshen Recipe (HAR) was formulated according to its method in treating CHD accompanied with insomnia in clinic. However, there are few studies related to the effect of HAR on myocardial injury and sleep disorders.* Purpose*. To investigate the effects of HAR on sleep deprivation- (SD-) induced myocardial I/R injury.* Methods*. Male Wistar rats receiving a daily gavage of HAR or vehicle were exposed to SD intervention while control rats had normal sleep. Then all rats were exposed to myocardial I/R. Hormone, vascular endothelial, and inflammatory related factors were detected before and after I/R, while cardiac injury, cardiac function, myocardial infarct size, and apoptosis were detected after I/R.* Results*. Levels of neuropeptide Y, vascular endothelial and inflammatory related factors were significantly increased while melatonin was decreased in vehicle-treated SD rats but not in HAR-treated SD rats after SD. In addition, cardiac injury, cardiac dysfunction, myocardial infarct size, and myocardial apoptosis were deteriorated in vehicle-treated SD rats but were ameliorated in HAR-treated SD rats after I/R.* Conclusion*. HAR not only improved SD-induced hormone disorders, inflammation, and endothelial dysfunction, but also alleviated I/R injury, which supports protective usage in CHD and psychocardiology.

## 1. Introduction

Insomnia and anxiety are associated with cardiovascular diseases and increase the risk for coronary heart disease (CHD) [1, 2], which is a hotspot in psychocardiology. Sleep deprivation (SD) plays an important role in the physiopathology of insomnia and anxiety, which is closely related to cardiovascular risk factors that aggravated CHD in pathogenesis and outcomes [3]. It is known that the deteriorated effect of SD has been associated with an increase in mortality and morbidity [4]. It is capable of inducing increased oxidative stress, inflammation, endothelial dysfunction, and accelerated atherosclerosis [3–6]. It has been reported that endothelin (ET-1) and proinflammatory cytokines are significantly elevated after SD, while TNF-alpha (TNF-*α*) is a primary mediator in triggering inflammation and apoptosis in myocardial injury [4, 5, 7]. In addition, deficiency of melatonin (MT) is one of the consequences of SD [8], while MT with anti-inflammatory and endothelium protective effects is involved in the modulation of cardiac dysfunction and myocardial ischemia/reperfusion (I/R) injury [9, 10]. Furthermore, SD is related to Bcl_2_ and Bax expression, and nuclear transcription factor (NF-*κ*B) signaling system, which plays a pathogenic role in inflammation and myocardial apoptosis [5, 11]. These factors could subsequently trigger cardiac injury. However, there is little research on hormone, inflammation, and apoptosis in the compound model of SD and myocardial I/R injury. Since experimental SD using platform method is an appropriate model for evaluation of cardiovascular complications of SD [12], we build compound model of SD and myocardial I/R injury using multiple platform method and coronary artery occlusion and reperfusion method.

Evidence shows that Traditional Chinese Medicine (TCM) has been devoted to alternative and complementary therapy for ischemic disease [13]. Chinese herbal medicine has been proved to be effective in antioxidation, anti-inflammation, antiapoptosis, protecting mitochondrial function, promoting angiogenesis, and so on [14]. Nowadays, it is widely accepted that multiple ingredients are responsible for the therapeutic effects of TCM. However, limited data are available for the effect of complex prescriptions of TCM on SD-induced myocardial I/R injury. HuoxueAnshen Recipe (HAR) has been used for symptomatic treatment of CHD in clinic and it is a modified formula with promoting blood circulation and tranquilization effects in TCM based on a traditional Chinese cardiotherapeutic prescription. Previous research has shown that HAR could significantly improve the morphological structures and functional abnormality induced by myocardial ischemia in rats through anti-inflammation [15]. The aim of this study was to investigate the effect of HAR on SD-induced hormone disorders, inflammation, and endothelial dysfunction, as well as exacerbating I/R injury and the potential mechanism.

## 2. Materials and Methods

### 2.1. Animals

The research has been conducted in accordance with the Use of Laboratory Animals by the U.S. National Institutes of Health. In this study, seventy-two male Wistar rats weighing approximately 270–290 g were obtained from the Vital River Laboratory Animal Technology Co. Ltd. [SCXX (Beijing) 2012-0001]. Animals were randomly divided into normal sleep control group and SD-intervention groups, including vehicle-treated SD group and HAR-treated SD group after three days in adaptation.

### 2.2. Sleep Deprivation

SD intervention involves the use of the modified multiple platform method [16, 17]. The apparatus consists of a water chamber (80 cm [diameter]) and 13 circular platforms (6 cm [height], 3 cm [diameter]), with 15 cm between each platform, submerged 1 cm below the water surface. The rats could move freely on the multiple platforms. When the animals slept, they touched or fell into the water because of the loss of muscle tone and woke up. The SD-intervention rats were maintained on a 24 h light per day for 7 days (maximum obvious pathological changes appeared after 7 days in our preexperiment [18]) at an ambient temperature of 23–25°C. Food and water bottles were located on a grid on top of the tank. The water was changed daily. The normal sleep control rats were housed under the same temperature space as the SD rats with a 12 : 12 h light-dark cycle.

### 2.3. Drug Preparation

HAR consists of four medicinal components (Table 1). All herbs were purchased from Beijing Fengtai Jinyuan Pharmaceutical Co., Ltd. (Beijing, China) and authenticated by researcher Guo Li-li, Research and Development center of TCM, Guang'anmen Hospital, China Academy of Chinese Medical Science, Beijing, China. Voucher specimens (Table 1) were deposited in the Herbarium of Guang'anmen Hospital, China Academy of Chinese Medical Science (Beijing, China).* Salvia miltiorrhiza Bunge, Astragalus mongholicus Bunge, and Ziziphus jujuba var. spinosa (Bunge) Hu ex H. F. Chow *were dried and mixed in proportion. The mixture was macerated 10 times (v/w) with 60% ethanol and was decocted twice for 1.5 h. The filtrates were mixed and condensed and then the* panax pseudoginseng Wall.* was added. These medicines were dried by vacuum-drier at 60°C and ground. The yield of HAR extract was 23.15% (w/w) according to the original herbs. The active compounds of HAR were determined referring to the Pharmacopeia of the People's Republic of China (Table 2). The resulting micropowder was suspended in 0.5% carboxymethylcellulose sodium solution to a final concentration of 1.4 g/kg (equal to the clinic dosage) for animal administration. HAR or vehicle (0.5% carboxymethylcellulose sodium (20141023) in high purity water) was given by gavage to rats in the HAR-treated SD and vehicle-treated SD groups, respectively, daily until the 7th day, 30 min before I/R.

### 2.4. I/R Heart Preparation

After 7 days, vehicle-treated SD rats and HAR-treated SD rats were anesthetized. Following endotracheal intubation, a ventilator was used to support their breathing (Chengdu Techman Technology Co. Ltd., China) [tidal volume 1.5 ml/100 g; respiratory rate 70 cycles/min]. Then the heart was exposed, and the left coronary artery was ligated with a 4-0 silk ligature over a 1 mm polyethylene tube (PE-10) for 50 min. Next, the coronary artery was reperfused for 2 h and 24 h, respectively, and myocardial I/R model was established. An electrocardiogram was performed to confirm the reliability of the animal model. Eight rats died during the operation period and ten rats died during postoperative period because of acute pumps failure or lethal arrhythmias.

### 2.5. Blood and Myocardium Analysis

Seven days after SD (before I/R) and 2 h after reperfusion (after I/R), blood was obtained from the abdominal aorta immediately prior to the removal of the heart and collected in serum and plasma separation tubes. Tubes were centrifuged at 3500 rpm at 4°C for 10 min and then serum, plasma, and heart were stored at −80°C, respectively, for further analyses. Heart homogenates were centrifuged at 15,000 rpm at 4°C for 30 min, and supernatants were used for measurements of TNF-*α*. The levels of plasma catecholamine (CA) and neuropeptide (NPY) were measured using enzyme-linked immunosorbent assay (ELISA) according to the procedures of ELISA kit (Shanghai Bluegene Biotech Co. Ltd., China). The levels of serum MT, C-reactive protein (CRP), and angiotensin II (AngII) were determined with ELISA kits (Cusabio Biotech Co. Ltd., China). The levels of serum ET-1 were determined with the endothelia radioimmunoassay kit (Beijing North Institute of Biological Technology, China). The levels of serum interleukin-6 (IL-6) and TNF-*α* were measured using ELISA kits (Multi Sciences (Lianke) Biotech Co. Ltd., China)

### 2.6. Measurement of Infarct Size

The left coronary artery was reoccluded at 24 h after reperfusion to determine the infarct size. Briefly, the hearts were perfused with 1% Evans blue and were sliced horizontally into five slices and then incubated in 1% TTC for 20 min at 37°C. Finally, the hearts were fixed in 4% paraformaldehyde. TTC stained ischemic tissue red but not the infarct area. The infarct areas (write color) and areas at risk (red color) were determined by planimetry with Image Pro Plus software (Media Cybernetics, USA) and measured infarct size was expressed as the percentage of the infarct area (IA)/area at risk (AAR).

### 2.7. Histological Analysis of Hematoxylin-Eosin (HE) Staining

Freshly dissected hearts at 2 h after reperfusion were fixed in 10% formalin and embedded in paraffin. The paraffin blocks were cut into 5 *µ*m slices and stained with HE. The pathological analysis was examined using an optical microscope (Olympus, Tokyo, Japan).

### 2.8. Cardiac Injury and Cardiac Function

Creatine kinase MB (CK-MB) and lactate dehydrogenase (LDH) were assayed at 2 h after reperfusion using their corresponding kits (Beckman Coulter Inc, USA). Transthoracic echocardiographic examinations were performed using Aloka 5000 digital color ultrasonic equipment (Japan). Before I/R and 2 h and 24 h after reperfusion, two-dimensional guide M-mode images of the short axis view of the left ventricle were carefully recorded. Ejection fraction (EF) was measured as previously described [19, 20].

### 2.9. Terminal Deoxynucleotidyl Transferase- (TdT-) Mediated dUTP-Digoxigenin Nick End-Labeling (TUNEL) Assay

At 2 h after reperfusion, the histochemical detection of apoptotic cells was determined by TUNEL staining on paraformaldehyde-fixed cardiac samples in paraffin blocks using an apoptosis assay kit (Roche Applied Science, Indianapolis, Ind). The apoptotic index was calculated as the number of TUNEL-positive myocytes to the total number of myocytes in 5 representative microscopic image views captured randomly using an IX70 Olympus microscope equipped with a camera (Japan).

### 2.10. Immunohistochemistry (IHC)

Heart samples were drawn at 2 h after reperfusion and detected as per manufacturer's instructions. Slides were blocked with 10% normal serum diluted 1 : 100 and incubated with rabbit anti-rat Bcl_2_, Bax, Caspase-3, and NF-*κ*B monoclonal antibodies (Abcam, Cambridge, UK) overnight at 4°C. Slides were washed three times in PBS for 3 min each and then incubated for 15 min at 37°C with a biotin-labeled, anti-rabbit detection antibody (Invitrogen, USA). Next, the slides were washed again and incubated with horseradish peroxidase-labeled streptavidin. Finally, the sections were then washed and developed with diaminobenzidine (DAB) reagent (Invitrogen, USA), counterstained with hematoxylin, and then dehydrated and mounted. The integrated optical density (IOD/area) in the captured fields was analyzed using Image Pro Plus software (Media Cybernetics, USA).

### 2.11. Western Blotting Analysis

Heart samples were drawn at 2 h after reperfusion and detected as previously described [17]. Frozen heart tissue was homogenized in lysis buffer. The protein concentration was determined using a BCA Protein assay kit (Cwbiotech, China). Next, 2 *µ*g/*µ*l of protein was separated using 10% SDS-PAGE and then electrophoretically transferred onto PVDF membranes (Millipore, USA). Proteins on the membranes were then probed using primary antibodies, including mouse Bcl_2_ (ab7973, abcam), Bax (ab32503, abcam), Caspase-3 (ab17185, abcam), NF-*κ*Bp65 (ab16502, abcam), and GAPDH (5174, CST). Following incubation with secondary antibodies, including goat anti-rabbit secondary antibody, the positive protein blots were developed using a chemiluminescent system, and the bands were quantified using the Gel Image system ver. 4.00 (Tanon, China). Data were shown as the percentage density of the blots.

### 2.12. Statistical Analysis

Values were expressed as the means ± standard deviation (S.D.). Statistical analysis was performed using SPSS 19.0 software (SPSS, Inc., Chicago, IL, USA). One-way ANOVA and Student's *t*-test were performed for analysis. The significance level was established at* P* < 0.05.

## 3. Results

### 3.1. Characterization of the Animals

**Figure 1:**
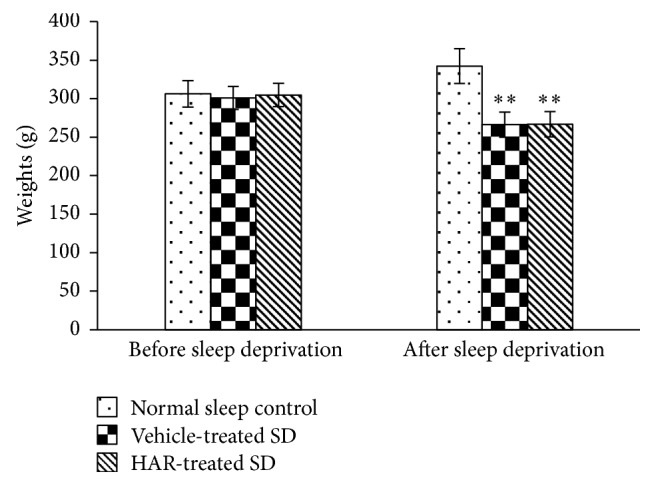
Effect of sleep deprivation on weights in rats. Values are means ± S.D. (*n* = 18). ^*∗∗*^*P* < 0.01 versus normal sleep control group.

SD rats showed significant mania and irritability with aggressive behavior and decreased body weight (*P* < 0.01), which indicated that the SD model was successfully established. HAR had no effect on the weight (Figure 1).

### 3.2. Hormone, Inflammation, and Endothelial Function

**Figure 2:**
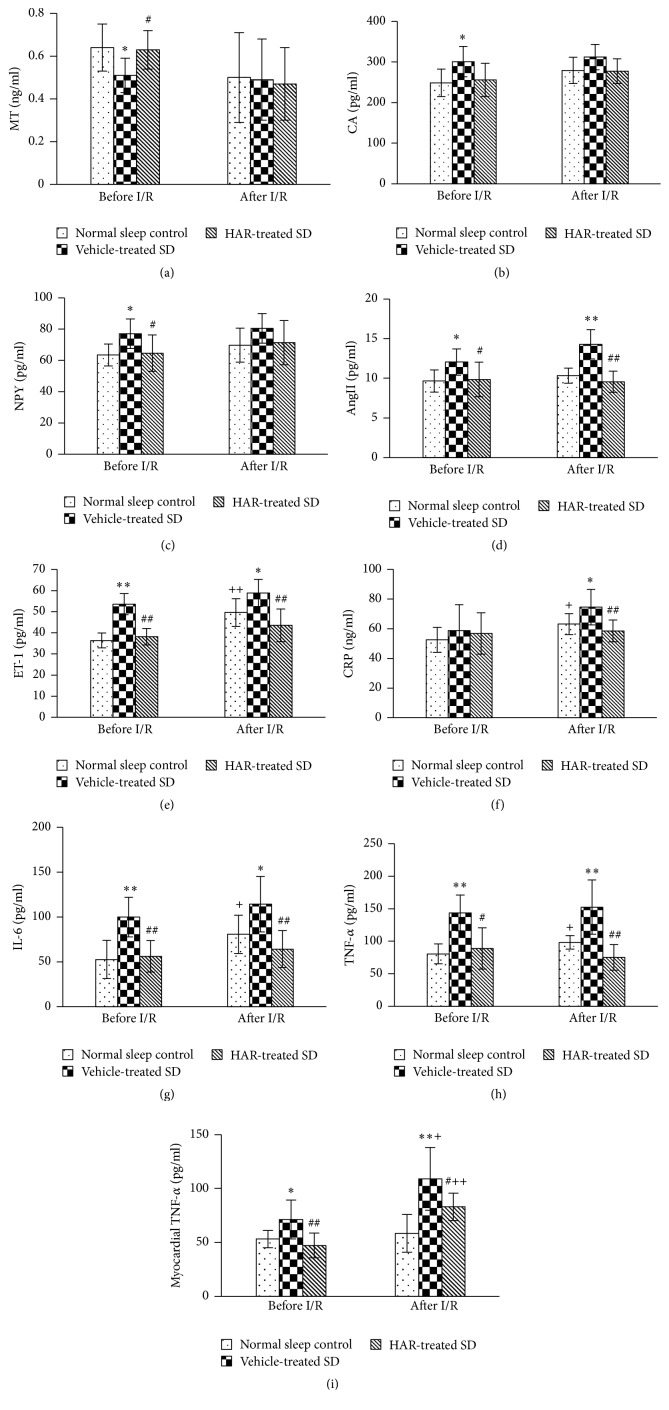
Effect of HuoxueAnshen Recipe on (a) serum MT, (b) plasma CA, (c) plasma NPY, (d) serum AngII, (e) serum ET, (f) serum CRP, (g) serum IL-6, (h) serum TNF-*α*, and (i) myocardial TNF-*α* before and 2 h after I/R. Values are means ± S.D. (*n* = 6). ^*∗*^*P* < 0.05, ^*∗∗*^*P* < 0.01 versus normal sleep control group; ^#^*P* < 0.05, ^##^*P* < 0.01 versus vehicle-treated SD group; ^+^*P* < 0.05, ^++^*P* < 0.01 versus related group before I/R.

The level of MT decreased while CA and NPY increased after SD (before I/R), but only the MT and NPY levels were improved in HAR-treated rats (*P* < 0.05) (Figures 2(a)–2(c)). In addition, the level of AngII, ET-1, IL-6, and TNF-*α* increased significantly before and after I/R, and CRP was elevated after I/R in vehicle-treated SD rats compared with the normal sleep control group (*P* < 0.05). However, HAR-treated SD group had lower levels (Figures 2(d)–2(h)). The level of myocardial TNF-*α* increased significantly in vehicle-treated SD group before I/R and further elevated after I/R (*P* < 0.05). However, the increase was less in the HAR-treated SD group compared with the vehicle-treated SD group (*P* < 0.05) (Figure 2(i)).

### 3.3. Infarct Size

**Figure 3:**
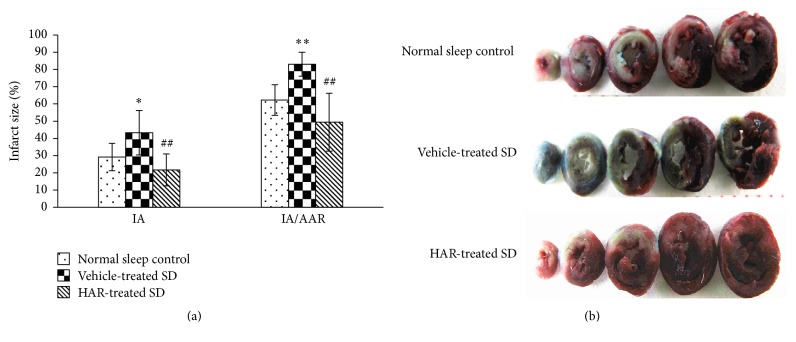
Effect of HuoxueAnshen Recipe on infarct size at 24 h after reperfusion. (a) IA and IA/AAR in all groups. Values are means ± S.D. (*n* = 6). ^*∗*^*P* < 0.05, ^*∗∗*^*P* < 0.01 versus normal sleep control group; ^##^*P* < 0.01 versus vehicle-treated SD group. (b) Representative histological section of cardiac tissue.

Compared with the normal sleep control group, the IA and IA/AAR were significantly increased in the vehicle-treated SD rats (*P* < 0.05) while improving in HAR-treated rats (*P* < 0.01) (Figure 3).

### 3.4. Cardiac Pathological Changes

**Figure 4:**
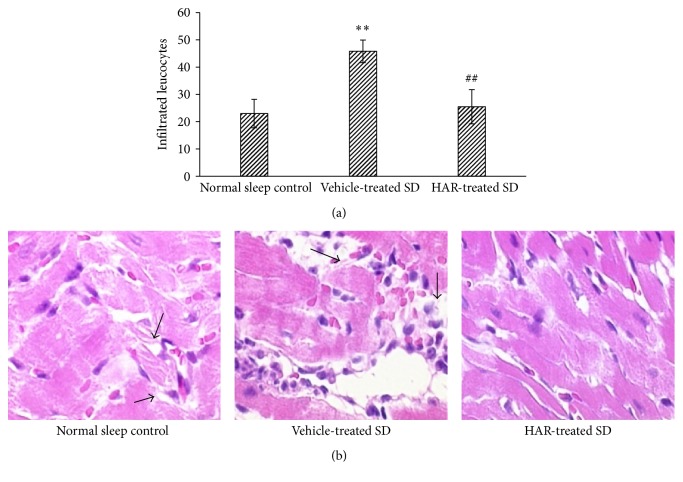
Effect of HuoxueAnshen Recipe on cardiac pathological changes at 2 h after reperfusion. (a) The number of infiltrated leucocytes. Values are means ± S.D. (*n* = 6). ^*∗∗*^*P* < 0.01 versus normal sleep control group; ^##^*P* < 0.01 versus vehicle-treated SD group. (b) Typical image (tissue sections were viewed at 40x magnification). The arrows refer to edema and infiltrated leucocytes.

Myocardial injury in vehicle-treated SD rats was more severe than that of the normal sleep control rats. There were vast areas of injury with more infiltrated leucocytes in vehicle-treated SD group (*P* < 0.01), while there were smaller areas of injury and fewer infiltrated leucocytes in HAR-treated SD group (*P* < 0.01). In the vehicle-treated SD group, there was severe loss of muscle fibers, fractured muscle bundle, edema, and infiltrated leucocytes. However, there was improvement in HAR-treated SD group (Figure 4).

### 3.5. Cardiac Injury and Cardiac Function

**Figure 5:**
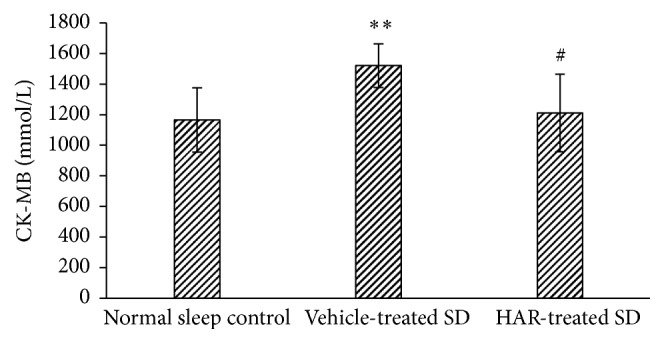
Effect of HuoxueAnshen Recipe on serum CK-MB at 2 h after reperfusion. Values are means ± S.D. (*n* = 6). ^*∗∗*^*P* < 0.01 versus normal sleep control group; ^#^*P* < 0.05 versus vehicle-treated SD group.

**Figure 6:**
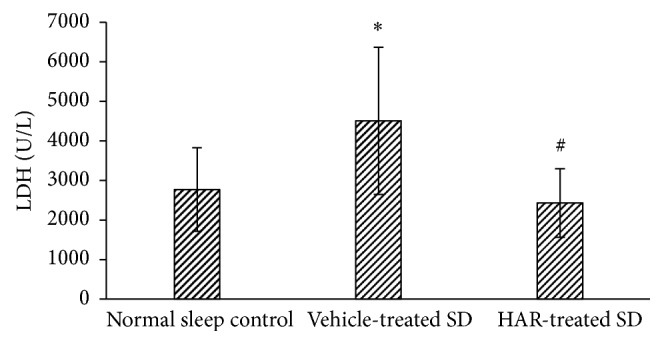
Effect of HuoxueAnshen Recipe on serum LDH at 2 h after reperfusion. Values are means ± S.D. (*n* = 6). ^*∗*^*P* < 0.05 versus normal sleep control group; ^#^*P* < 0.05 versus vehicle-treated SD group.

**Figure 7:**
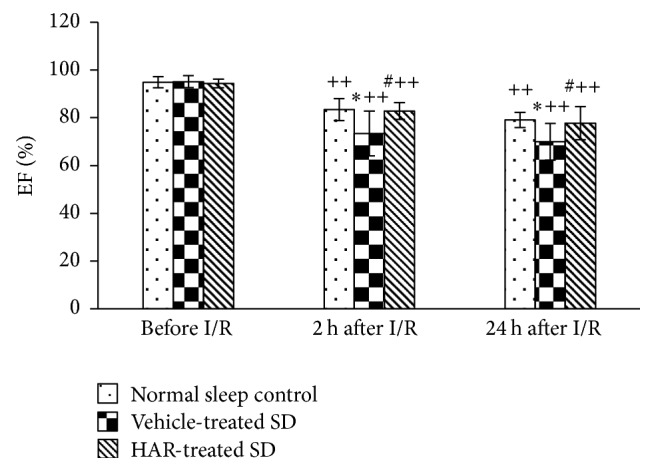
Effect of HuoxueAnshen Recipe on ejection fraction (EF) at 2 h and 24 h after reperfusion. Values are means ± S.D. (*n* = 6). ^*∗*^*P* < 0.05 versus normal sleep control group; ^#^*P* < 0.05 versus vehicle-treated SD group; ^++^*P* < 0.01 versus related group before I/R.

The levels of CK-MB and LDH increased while EF decreased in vehicle-treated SD group, but not in the HAR-treated SD group (*P* < 0.05), which reflected the improvement in cardiac recovery (Figures 5–7).

### 3.6. Myocardial Apoptosis

**Figure 8:**
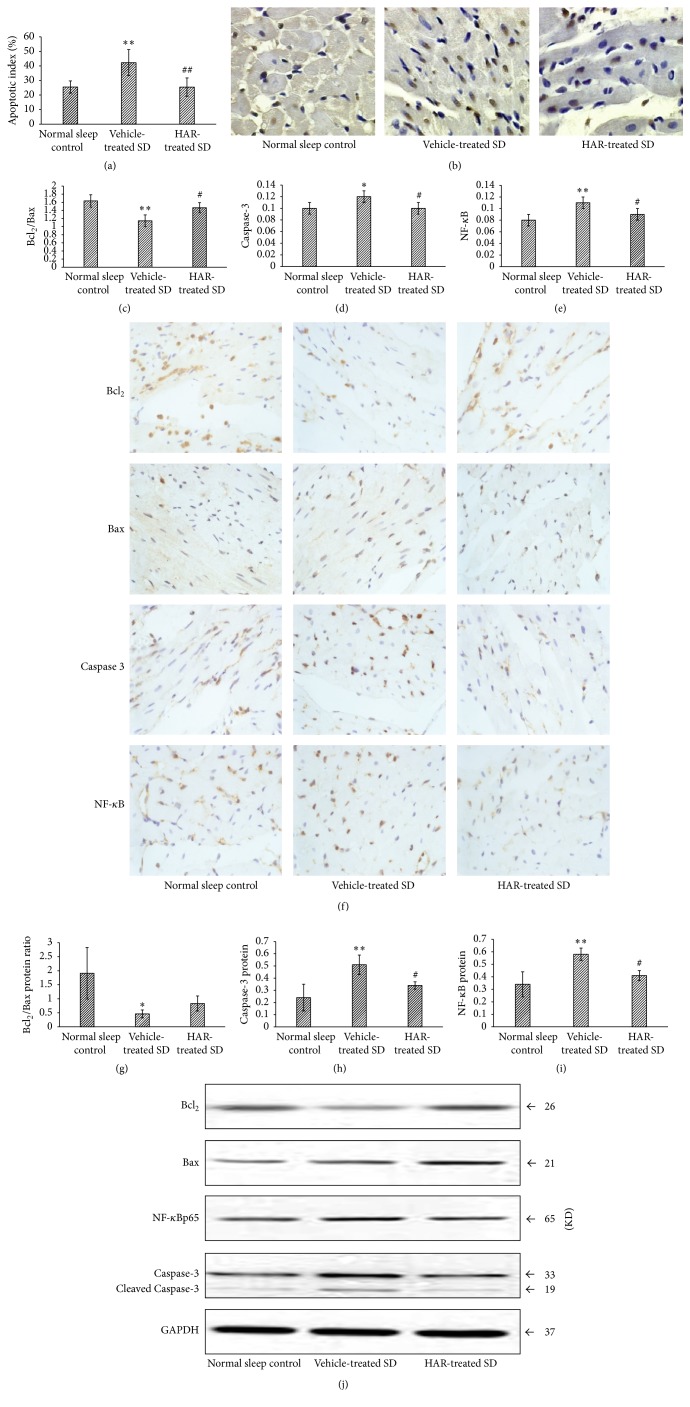
Effect of HuoxueAnshen Recipe on myocardial apoptosis at 2 h after reperfusion. (a) The apoptotic index and (b) typical image (tissue sections were viewed at 40x magnification) by TUNEL staining. The expression of (c) Bcl_2_/Bax ratio, (d) Caspase-3, (e) NF-*κ*B, and (f) typical image (tissue sections were viewed at 40x magnification) by IHC staining. The expression of (g) Bcl_2_/Bax ratio, (h) Caspase-3, (i) NF-*κ*B, and (j) typical image by western blot. Values are means ± S.D. (*n* = 6). ^*∗*^*P* < 0.05, ^*∗∗*^*P* < 0.01 versus normal sleep control group; ^#^*P* < 0.05, ^##^*P* < 0.01 versus vehicle-treated SD group.

There was a higher cardiac myocyte apoptotic index in vehicle-treated SD group compared to normal sleep control group but not in the HAR-treated SD group (*P* < 0.01) (Figures 8(a) and 8(b)). In IHC method (Figures 8(c)–8(f)), the ratio of the Bcl_2_/Bax decreased and expression of Caspase-3 and NF-*κ*B increased in SD rats compared with normal sleep control rats, while HAR resulted in a balance of these expression patterns (*P* < 0.05). In western blot analysis (Figures 8(g)–8(j)), the ratio of Bcl_2_/Bax protein was markedly lowered in vehicle-treated SD group whereas a smaller decrease in HAR-treated SD group, and the expression of Caspase-3 and NF-*κ*B protein increased significantly in vehicle-treated SD group while it was less in HAR-treated SD group (*P* < 0.05).

## 4. Discussion

This study demonstrates that HAR could not only regulate hormone (NPY and MT) and improve vascular endothelial related factors (AngII and ET-1) and inflammatory related factors (IL-6 and TNF-*α*) after SD (before I/R), but also decrease infarct size, myocardial enzyme, and expression of NF-*κ*B and Caspase-3 while increasing EF and the Bcl_2_/Bax ratio after SD + I/R (after I/R). Previous study investigated the effect of HAR on acute myocardial infarction [15], which had not involved insomnia, anxiety, and sleep disorders, and it had not found the tranquilization effect of HAR. Therefore, HAR inhibited SD-induced myocardial I/R injury, which provided an alternative and complementary therapy for psychocardiology.

Results have shown that HAR had preventive and protective effects. It has been recognized that SD is associated with cardiovascular pathological factors, and it is an important stress which could induce autonomic nervous dysfunction, neuroendocrine dysfunction, oxidative stress, inflammation, and endothelial dysfunction [3, 4]. Therefore, reducing SD-induced hormone disorders and cardiovascular pathological factors is an important preventive way. In our study, NPY, CA, AngII, ET-1, CRP, IL-6, and TNF-*α* increased while MT decreased after SD, which is characteristic of the beginning of myocardial injury due to hormone disorders, inflammation, and endothelial dysfunction. While HAR could regulate hormone by decreasing NPY level and increasing MT level, it further reduces pathological factors through alleviating inflammatory injury and endothelial dysfunction, which provided preventive effects for latter I/R injury.

Results also have shown that HAR had cardioprotective effects. It is known that TNF-*α* can simultaneously activate apoptosis [21], and high concentrations of TNF-*α* may induce the activation of NF-*κ*B; NF-*κ*B can further activate inflammatory cytokines and apoptosis-related factors in I/R injury [22–25]. Therefore, inhibiting the NF-*κ*B signaling system to reduce inflammation and apoptosis is an important way. In our study, inflammation, endothelial dysfunction, and apoptosis were severe after SD + I/R, while HAR could inhibit the NF-*κ*B signaling pathway, alleviate inflammation and apoptosis, and further improve myocardial injury.

Importantly, MT had the protective therapeutic effect on promoting plaque stability, reducing apoptosis, and lowering myocardial enzyme and infarct size after I/R in sleep disorder rats [26]. We found that HAR could increase MT level, which may have preventive therapeutic effects on heart, and that the mechanism may be involved in anti-inflammatory, endothelium protective, and antiapoptosis effects in I/R injury. Thus, MT may play a role in bridging the regulating hormone and alleviating myocardial injuries [10]. However, the protective effect of HAR on the signaling pathway involved in the regulation of MT levels and NF-*κ*B expression requires further investigations.

Despite TCM being not sufficient, it has successfully been used for centuries to treat various ailments and it is attractive for its anti-inflammation, antioxidation, antiapoptosis, and mitochondrial function protection effects in the treatment of CHD [13, 14]. In our study, effects of HAR could be manifested by regulating hormone disorders, anti-inflammation, and antiapoptosis, which proved the cardioprotective effects of HAR on SD-induced I/R injury by multiple targets and multiple paths. At present, it has been reported that TNF-*α* antagonism is most likely not beneficial in the treatment of acute myocardial infarction because it decreases systemic inflammation while increasing platelet activation, which does not affect peripheral vasomotor or fibrinolytic function [27]. This finding indicated that single-target drug may be associated with side effects. Since SD-induced aggravated I/R injury contributes to various pathological changes, which might be a network, single-target drug may not work, while HAR could improve the pathological factors by multiple ways in the treatment of CHD accompanied with insomnia and anxiety, which will have prospects for development.

## 5. Conclusions

HAR could not only improve hormone disorders, inflammation, endothelial dysfunction, and mental state after SD, but also ameliorate SD-induced myocardial apoptosis and I/R injury. It is shown that HAR could improve cardiac injury, and, more importantly, it had notable effect on cardiac injury combined with sleep disorders. Briefly, HAR could be therapeutically used in the prevention of SD-induced myocardial I/R injury and the NF-*κ*B signaling system could be a potential mechanism. Moreover, the cardioprotective effect of HAR against SD-induced myocardial I/R injury supports the prophylactic usage of HAR in CHD accompanied with insomnia and anxiety, which may provide a new promising potential therapeutic alternative for psychocardiology.

## Figures and Tables

**Table 1:** Recipe of HAR formulation.

Components	Voucher number	Part used	Place of production	Season	Processing	Amount used (g)
Salvia miltiorrhiza Bunge	20100806	Root	Hebei	Winter	Dried in the sun and used crudely	30
Panax pseudoginseng Wall.	20100011	Root	Yunnan	Autumn	Exposed to the sun and used crudely	6
Astragalus mongholicus Bunge	20100478	Root	Inner Mongolia	Autumn	Sliced and dried with removal of head and fine roots	10
Ziziphus jujuba var. spinosa (Bunge) Hu ex H. F. Chow	20100582	Seed	Hebei	Autumn	Dried in the sun and used crudely	15

**Table 2:** Active compounds of HAR.

Effective constituent	Content%
Salvianolic acid B	35.68
Tanshinone IIA	13.97
Notoginsenoside R1	0.20
Ginsenoside Rg1	1.01
Ginsenoside Re	0.11
Ginsenoside Rb1	0.93
Jujuboside A	0.11
Jujuboside B	0.18
Astragaloside	0.046
